# Advanced Electronic Packaging Technology: From Hard to Soft

**DOI:** 10.3390/ma16062346

**Published:** 2023-03-15

**Authors:** Yue Gu, Yongjun Huo

**Affiliations:** 1 School of Materials Science and Engineering, Beijing Institute of Technology, Beijing 100081, China; 2 Department of Neurosurgery, Yale University, New Haven, CT 06520, USA

Packaging is a pivotal step in electronic device manufacturing, determining the translational performance of bare chips. Nowadays, the landscape has been changing in the electronic packaging society due to the fact that manufacturing technologies for silicon have reached a physical size limit. Upon the dawn of the post-Moore’s law era, researchers must steer their directions to find alternative materials or structures, e.g., carbon nanotubes/graphene [1,2] and heterogenous integration [3], to either continue Moore’s law or surpass it (See Table 1). Numerous emerging technologies, such as power electronics and LiDAR for automobiles [4], have already become fast-growing and significant players in the semiconductor industry. Related packaging technologies must follow the pace of chip development to meet the increasing needs for high-speed and high-bandwidth computation in future devices.

Currently, the total number of micro-bumps in a single advanced integrated circuit (IC) chip has already surpassed 100,000, and the pitch size between adjacent microbumps can be lower than 20 μm. Therefore, the continuing pursuit of bonding technology [5] is driven by the down-scaling trend of microbumps with an ultra-fine pitch for a more compact and reliable design of 3D packaging architectures. The mainstream industry has adopted through-silicon via (TSV) technology by utilizing copper (Cu) pillars with lead-free solder as micro-bumps to achieve 3D IC packaging or heterogenous integration as both electrical and mechanical interconnections. However, with the downward scaling of micro-bumps’ size, the fraction of Cu–Sn intermetallic compounds (IMCs) has increased drastically due to the higher inter-diffusion rate triggered by a through-surface diffusion pathway [6], resulting in lower electrical conductance and severe reliability issues in traditional micro-bumps for 3D IC packaging. As a technical solution, metal-based solid-state direct bonding has been conceived as the next-generation integration technology to completely eliminate the formation of IMCs from the joint interfaces. However, metal-based solid-state direct bonding usually suffers from the poor quality of its bonding interface with voids and defects, mainly due to the ubiquitous asperities on metal surfaces. Typically, as the bonding temperature/pressure increases, the bonding quality can be improved. For example, a high-strength Cu-to-Cu direct bonding can be achieved when utilizing a high temperature and a high pressure under a high vacuum. However, the usage of high temperature/pressure must be avoided in packaging interconnections to mitigate the thermo-mechanical stress level or pressure-induced micro-crack generation. Theoretically, the atomic-level direct contact is the prerequisite to realize solid-state bonding with high reliability [7]. Surface asperities can be overcome by utilizing a thermo-plastically deformation mechanism, whereby increasing the percentage of the atomic-level direct contact area. In essence, it is important to design and modulate the properties of solid-state bonding materials [8], such as yield strength, microstructure, orientations, and surface state, in order to further reduce bonding temperature/pressure. To date, it is highly desirable to develop a suitable metal-based solid-state direct bonding method with low bonding temperature/pressure for microelectronic packaging industry [9].

In the meantime, more efforts are being spent on leveraging the improved functions of present semiconductor chips in consumer electronic products, which currently have not fulfilled the extremized performance of advanced chips due to the unresolved issues of traditional packaging. One good example is the intrinsic properties of silicon carbide (SiC) semiconducting material [10] have promised a fantasy that an electronic device can sustain a high working temperature up to 350 °C, but it has failed to industrially realize such promise simply because of the lack of suitable packaging materials. Nevertheless, recent development in packaging technology, such as transient liquid phase (TLP) bonding [11] or nanoparticle sintering process [12], kindles the hope for the entire field of high-temperature electronics. Silver has excellent electrical and thermal conductivity, good mechanical properties, and chemical stability, all of which come at a reasonable cost, making it a popular choice for electronic packaging. Sintering technology for silver nanoparticles (AgNPs) is a silver-based bonding method, which has become popular among academic and industrial packaging communities in recent years. When metal particles are reduced to the nanoscale, they become highly unstable due to the large ratio of surface area to volume. To prevent agglomeration, organic coatings are used to encapsulate metallic nanoparticles in a dispersion solvent. The nanosintering process requires only a small amount of thermal energy to decompose the coatings and solvent, allowing the metal particles to spontaneously connect with each other through agglomeration. Additionally, the surface diffusion mechanism of nanoscale particles can significantly lower the melting point of surface atoms, facilitating the bonding process at much lower temperatures than the bulk metal material’s melting point. In contrast, transient liquid phase bonding, also known as solid–liquid interdiffusion bonding (SLID), involves the design of a “high melting point-low melting point” metallurgical combination for low-temperature bonding. At a low temperature, the low-melting-point metal turns into a molten phase and wets the surface of the high-melting-point metal at the beginning of the bonding process. During subsequent solid–liquid interdiffusion, the high-melting-point metal reacts with the low-melting-point metal in the liquid phase, causing the ratio of metal elements at the bonding joint interface to change continuously and leading to phase transformation. Under isothermal conditions, the liquid phase solidifies, forming a metallurgical bonding joint. The new phases formed at the bonding joint interface can be fully homogenized and stabilized through sufficient diffusion, resulting in melting points that are much higher than that of the original low-melting-point metal. Therefore, transient liquid phase bonding technology offers the advantage of a low-temperature process for high-temperature applications. Consequently, both AgNP sintering interconnection and TLP bonding technology have been considered as great candidates for high-temperature electronic packaging in future development. More broadly, for the development of mainstream “Hard” packaging technology, that is, “More Moore” or “More-than-Moore”, gives us opportunities to explore the unknown with non-generic packaging designs for integrated circuit (IC), micro-electro-mechanical system (MEMS), optoelectronics, lasing photonics, and power electronics, among others.

On the other hand, with many outstanding applications, wearable electronic devices have drawn more attention in the academia since humans care more about their health and life qualities [13], and the conformable interfacing between humans and electronic devices becomes a primary quest. According to a search on the Web of Science database, the number of publications with soft electronics in the title, abstract, or keywords has increased from ~100 in 2010 to over 3000 in 2021. This trend is expected to continue as the field of soft electronics continues to develop and new applications are discovered.

The definition of wearable electronic devices has two folds. One is the simple definition of an electronic device that can be worn, such as an electronic watch. The other means an electronic device with a much improved conformability with the human body surfaces, like a bandage. Some of the notable developments in electronic packaging for wearable devices and soft electronics include (1) stretchable and flexible substrates, which are the base materials that electronic components are mounted on, such as polyimide and elastomers; (2) encapsulation materials, which are the protection matrix to embed electronic components, such as silicone and polyurethane; and (3) additive manufacturing techniques, such as 3D printing and inkjet printing, which allow for the creation of complex geometries that are electronically functional. The first category of wearable devices does not relate much to the advancements in packaging materials or technologies, so it is beyond the scope of this article. We will primarily discuss the second category in the remainder of this article.

One motivation for developing soft wearable devices is that they can sense vital biological information from the human body [14]. The low mechanical modulus of these devices enables them to establish an intimate and unobstructed contact with the biological surface, thereby enhancing the efficiency of tissue–device coupling [15]. At this interface, numerous benefits have emerged, such as minimizing energy loss, optimizing physiological signal collection, reducing capacitive coupling of electromagnetic noise or motion artifacts, and enabling continuous long-term operation. In recent years, the transformation of well-developed rigid sensors and actuators into soft forms has been a widely pursued methodology to build soft electronics. Two major approaches are the integration of rigid electronic components in a soft scaffold, such as silicone, and the synthesis of intrinsic flexible or stretchable materials, such as a conductive polymer [16]. Table 2 summarizes current designs for achieving softness.

The first approach takes advantage of mature electronic packaging and microfabrication techniques in the semiconductor industry and a large variety of applicable materials, making the engineered devices more versatile and robust. We can envision that with well-developed packaging materials and technologies, we can easily convert transistors with high density and small feature size into soft and wearable forms. The primary challenge during packaging is to overcome the weak interfaces between rigid electronic components and the soft scaffold material. Mechanical and further electrical failures mostly occur at these interfaces, dramatically shortening the lifetime of these devices, especially in the scenarios of great bending, folding, or stretching of the devices. Resolutions to tackle this issue include embedding an intermediate layer that employs the mechanical modulus between the rigid and soft parts, thereby acting as a middle stepper. Another means is to utilize ultrasound at the interface to facilitate the interdiffusion of the two materials and form covalently bonded interfaces [28]. New functional packaging materials, as represented by intrinsic polymeric materials, could eliminate the mechanical mismatch problem. However, these materials’ robustness and properties are incompatible with traditional materials, such as silicon. To summarize, pursuing this aim largely relies on the innovation of nature-like soft, adaptive, and collaborative sensors and actuators and the development of soft and functional materials.

Recent advances in soft devices and machines with flexible structures or integrated sensing ability have shown a great potential to interact with the surrounding environments, including the human body, safely and imperceptibly. However, most reported devices have only one built-in modality, which significantly limits their clinical applications [29]. One important reason is the difficulty in packaging a more complex electronic system. The reliability of such a system under deformation could drop noticeably. Another reason is the lack of interconnecting technologies between different wearable devices or systems. Most present packaging technologies have not emphasized such interconnection. Overall, there is still a significant gap in realizing the so-called intelligent soft bio-sensing and actuation systems. The next-generation soft wearable electronic devices are expected to embed multiple sensors and show the capability of capturing different physiological signals. This makes electronic packaging an enabling technology to revolutionize engineering applications, such as environmental monitoring, human–machine interaction, and biomedical engineering for disease diagnosis and therapeutic functions.

In summary, although emerging technologies might be quite different when electronic devices turn “soft”, the essence of electronic packaging technology will always focus on the interconnection, integration, and protection of associated microsystems. To quote Richard Feynman [30], for both “Hard” and “Soft” functional devices, there is still plenty of room at the bottom for advancing electronic packaging technology.

## Figures and Tables

**Table 1:** Recent milestones in IC development: feature sizes and packaging structures.

Developed Time (Year)	Feature Size (nm)	Device Architecture	Packaging Structure
2004	90	Strained silicon/germanium	First TSV-enabled homogenous die Stack device (2004)
2004	65	Enhanced strain silicon	
2006	45	Hi-k metal gate silicon	Homogenous 3D-stacked memory with TSVs (Samsung, 2006)
2008	32	Enhanced Hi-k metal gate silicon	
2008	22 (20 or 24)	FinFET and silicon	Chip-on-wafer-on-substrate (CoWoS) 2.5D packaging (Xilinx, 2012)
2014–2018	14, 10, or 7	Enhanced FinFET	
2020–2021	5 or 2	Gate-all-around FET/nanosheet	Embedded multi-die interconnect bridge (EMIB) structure (Intel, 2014)
2021	7	Vertical-transport FET	
2010–2022	5, 4, or 2	Carbon nanotube FET	3D chiplet integration (Intel, 2019)
2022	<1	Graphene FET	

**Table 2:** Summary of designs for achieving softness in wearable electronics.

Approach	Design	Reference
Extendable interconnections between rigid electronic components	Island-bridge serpentine	[17]
	Kirigami	[18]
	Origami	[19]
	Wave/wrinkle	[20]
	Mesh/textile	[21]
	Crack	[22]
	Interlock	[23]
Intrinsic soft materials and systems	Polymer	[24]
	Liquid metal	[25]
	Hydrogel	[26]
	Embedded nanomaterials	[27]
